# 9-(4-Hy­droxy-3,5-dimeth­oxy­phen­yl)-3,3,6,6-tetra­methyl-3,4,5,6,7,9-hexa­hydro-1*H*-xanthene-1,8(2*H*)-dione

**DOI:** 10.1107/S1600536812010410

**Published:** 2012-03-14

**Authors:** V. Sughanya, N. Sureshbabu

**Affiliations:** a Department of Chemistry, Annamalai University, Annamalai nagar 608 002, Tamil Nadu, India

## Abstract

In the title compound, C_25_H_30_O_6_, the two fused cyclo­hexa­none rings have envelope conformations, whereas the central pyran ring is roughly planar [mximum deviation = 0.045 (2) Å]. The pyran and benzene rings are almost perpendicular to each other, making a dihedral angle of 86.32 (2)°. In the crystal, molecules are linked *via* pairs of O—H⋯O hydrogen bonds, forming inversion dimers.

## Related literature

For the synthesis of xanthenes, see: Vang & Stankevich (1960 ▶); Hilderbrand & Weissleder (2007 ▶). For their pharmaceutical properties, see: Lambert *et al.* (1997 ▶); Poupelin *et al.* (1978 ▶); Hideo (1981 ▶); Selvanayagam *et al.* (1996 ▶); Jonathan *et al.* (1988 ▶). For related structures, see Mehdi *et al.* (2011 ▶); Odabasoglu *et al.* (2008 ▶). For the assignment of ring conformations, see: Cremer & Pople (1975 ▶).
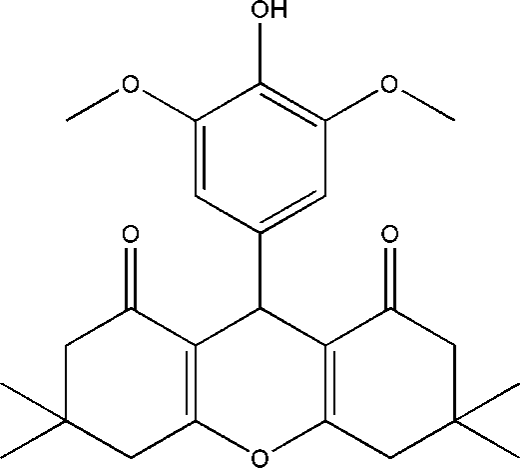


## Experimental

### 

#### Crystal data

C_25_H_30_O_6_
*M* *_r_* = 426.49
Triclinic, 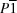

*a* = 9.4268 (9) Å
*b* = 10.2468 (10) Å
*c* = 12.6122 (11) Å
α = 84.973 (6)°
β = 70.377 (5)°
γ = 75.676 (6)°
*V* = 1111.83 (18) Å^3^
*Z* = 2
Mo *K*α radiation
μ = 0.09 mm^−1^
*T* = 295 K
0.30 × 0.25 × 0.20 mm

#### Data collection

Bruker Kappa APEXII CCD diffractometer
Absorption correction: multi-scan (*SADABS*; Bruker, 2004 ▶) *T*_min_ = 0.924, *T*_max_ = 0.982
20849 measured reflections
5233 independent reflections
2876 reflections with *I* > 2σ(*I*)
*R*_int_ = 0.052

#### Refinement

*R*[*F* ^2^ > 2σ(*F* ^2^)] = 0.051
*wR*(*F* ^2^) = 0.157
*S* = 0.98
5233 reflections
288 parameters
H-atom parameters constrained
Δρ_max_ = 0.28 e Å^−3^
Δρ_min_ = −0.21 e Å^−3^

### 

Data collection: *APEX2* (Bruker, 2004 ▶); cell refinement: *APEX2* and *SAINT* (Bruker, 2004 ▶); data reduction: *SAINT* and *XPREP* (Bruker, 2004 ▶); program(s) used to solve structure: *SIR92* (Altomare *et al.*, 1993 ▶); program(s) used to refine structure: *SHELXL97* (Sheldrick, 2008 ▶); molecular graphics: *ORTEP-3* (Farrugia, 1997 ▶) and *Mercury* (Macrae *et al.*, 2008 ▶); software used to prepare material for publication: *SHELXL97*.

## Supplementary Material

Crystal structure: contains datablock(s) global, I. DOI: 10.1107/S1600536812010410/im2356sup1.cif

Structure factors: contains datablock(s) I. DOI: 10.1107/S1600536812010410/im2356Isup5.hkl

Supplementary material file. DOI: 10.1107/S1600536812010410/im2356Isup3.cml

Additional supplementary materials:  crystallographic information; 3D view; checkCIF report

## Figures and Tables

**Table 1:** Hydrogen-bond geometry (Å, °)

*D*—H⋯*A*	*D*—H	H⋯*A*	*D*⋯*A*	*D*—H⋯*A*
O5—H5⋯O2^i^	0.82	2.02	2.762 (2)	151

Symmetry code: (i) 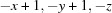.

**Fig. 1.**
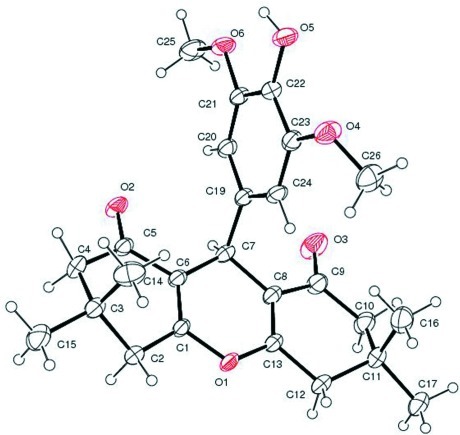
: Molecular structure of (I), showing displacement ellipsoids at the 30% probability level.

**Fig. 2.**
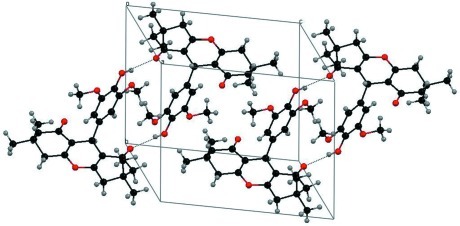
: Packing diagram for (I) showing the formation of O—H···O hydrogen bonds between the molecules in the unit cell.

## supplementary crystallographic information

### Comment

Xanthene is the parent compound of a number of naturally occurring substances and some synthetic dyes. Xanthene derivatives are used as dyes (Hilderbrand & Weissleder, 2007) and they possess biological properties like antibacterial, antiviral, anti-inflammatory (Jonathan *et al.*, 1988) activities and are therefore used in medicine. Ehretianone, a quinonoid xanthene was reported to possess antisnake venom activity (Selvanayagam *et al.*, 1996; Lambert *et al.*, 1997; Poupelin *et al.*, 1978; Hideo, 1981).

In the title compound (I), the cyclohexenone rings C1–C6 and C8–C13 both adopt an envelope conformation. In contrast, the pyran ring (O1/C1/C6/C8/C13) is almost planar with a slight deviation of C7 (0.99 Å) from the (C8/C13/O1/C1/C6) plane. The pyran ring and phenyl ring are almost perpendicular to one another making a dihedral angle of 86.32 (2)°. The bond lengths and angles are consistent with the reported structure (Odabasoglu *et al.*, 2008; Mehdi *et al.*, 2011). In the crystal structure, a relatively short intermolecular O5—H5···O2 hydrogen bond leads to the observation of centrosymmetrical dimers.

### Experimental

The title compound was prepared in two stages (Vang & Stankevich, 1960). In the first stage, a mixture of 4-hydroxy-3,5-dimethoxybenzaldehyde (0.5 g, 8 m mol), 5,5-dimethylcyclohexane-1,3-dione (1.15 g, 1.6 mmol) and 10 ml of ethanol was heated to 70°C for about 10 minutes. The reaction mixture was allowed to cool to room temperature and the resulting solid intermediate 2,2'-((4-hydroxy-3,5-dimethoxyphenyl)methylene)bis(3-hydroxy-5,5-dimethylcyclohex-2-enone) was filtered and dried. In the second stage, about 0.5 g of this intermediate were dissolved in 25 ml of ethanol. The content was refluxed together with 15 drops of concentrated hydrochloric acid for 30 minutes with the reaction being monitored by TLC. After completion of the reaction, the reaction mixture was poured into crushed ice and stirred well. The solid separated was filtered, dried and then recrystallized from ethanol to yield colourless crystals of t he title compound (m.p. 490–492 K; yield: 85%).

### Refinement

All hydrogen atoms of the title compound were identified from the difference electron map and subsequently treated as riding atoms with distances of d(C–H) = 0.96 Å (for CH_3_) with *U*_iso_(H) = 1.5 *U*_eq_(C), d(C–H) = 0.97 Å (for CH_2_) with *U*_iso_(H) = 1.2 *U*_eq_(C), d(C–H) = 0.98 Å (for CH) with *U*_iso_(H) = 1.5 *U*_eq_(C) and d(C–H) = 0.93 Å (for aromatic CH) with *U*_iso_(H) = 1.2 *U*_eq_(C). The hydroxyl hydrogen atom was also identified from the difference electron map and was allowed to ride on the parent O atom with d(O–H) = 0.82 Å and *U*_iso_(H) = 1.5 *U*_eq_(O).

### Crystal data

**Table:**

C_25_H_30_O_6_	*Z* = 2
*M**_r_* = 426.49	*F*(000) = 456
Triclinic, *P*1	*D*_x_ = 1.274 Mg m^−^^3^
Hall symbol: -P 1	Mo *K*α radiation, λ = 0.71073 Å
*a* = 9.4268 (9) Å	Cell parameters from 5637 reflections
*b* = 10.2468 (10) Å	θ = 2.4–27.5°
*c* = 12.6122 (11) Å	µ = 0.09 mm^−^^1^
α = 84.973 (6)°	*T* = 295 K
β = 70.377 (5)°	Block, colourless
γ = 75.676 (6)°	0.30 × 0.25 × 0.20 mm
*V* = 1111.83 (18) Å^3^	

### Data collection

**Table:**

Bruker Kappa APEXII CCD diffractometer	5233 independent reflections
Radiation source: fine-focus sealed tube	2876 reflections with *I* > 2σ(*I*)
Graphite monochromator	*R*_int_ = 0.052
ω and φ scan	θ_max_ = 28.3°, θ_min_ = 2.4°
Absorption correction: multi-scan (*SADABS*; Bruker, 2004)	*h* = −12→12
*T*_min_ = 0.924, *T*_max_ = 0.982	*k* = −13→13
20849 measured reflections	*l* = −16→16

### Refinement

**Table:**

Refinement on *F*^2^	Secondary atom site location: difference Fourier map
Least-squares matrix: full	Hydrogen site location: inferred from neighbouring sites
*R*[*F*^2^ > 2σ(*F*^2^)] = 0.051	H-atom parameters constrained
*wR*(*F*^2^) = 0.157	*w* = 1/[σ^2^(*F*_o_^2^) + (0.0666*P*)^2^ + 0.3374*P*] where *P* = (*F*_o_^2^ + 2*F*_c_^2^)/3
*S* = 0.98	(Δ/σ)_max_ < 0.001
5233 reflections	Δρ_max_ = 0.28 e Å^−^^3^
288 parameters	Δρ_min_ = −0.21 e Å^−^^3^
0 restraints	Extinction correction: *SHELXL97* (Sheldrick, 2008), Fc^*^=kFc[1+0.001xFc^2^λ^3^/sin(2θ)]^-1/4^
Primary atom site location: structure-invariant direct methods	Extinction coefficient: 0.008 (2)

### Special details

**Table:**

Geometry. All e.s.d.'s (except the e.s.d. in the dihedral angle between two l.s. planes) are estimated using the full covariance matrix. The cell e.s.d.'s are taken into account individually in the estimation of e.s.d.'s in distances, angles and torsion angles; correlations between e.s.d.'s in cell parameters are only used when they are defined by crystal symmetry. An approximate (isotropic) treatment of cell e.s.d.'s is used for estimating e.s.d.'s involving l.s. planes.
Refinement. Refinement of *F*^2^ against ALL reflections. The weighted *R*-factor *wR* and goodness of fit *S* are based on *F*^2^, conventional *R*-factors *R* are based on *F*, with *F* set to zero for negative *F*^2^. The threshold expression of *F*^2^ > σ(*F*^2^) is used only for calculating *R*-factors(gt) *etc*. and is not relevant to the choice of reflections for refinement. *R*-factors based on *F*^2^ are statistically about twice as large as those based on *F*, and *R*- factors based on ALL data will be even larger.

### Fractional atomic coordinates and isotropic or equivalent isotropic displacement parameters (Å^2^)

**Table:**

	*x*	*y*	*z*	*U*_iso_*/*U*_eq_	
C1	0.2424 (2)	0.1023 (2)	0.33354 (14)	0.0336 (5)	
C2	0.1297 (3)	0.0482 (2)	0.30200 (15)	0.0418 (5)	
H2A	0.0310	0.0657	0.3621	0.050*	
H2B	0.1666	−0.0486	0.2923	0.050*	
C3	0.1076 (3)	0.1131 (2)	0.19256 (16)	0.0466 (6)	
C4	0.2682 (3)	0.0985 (3)	0.10508 (16)	0.0509 (6)	
H4A	0.3068	0.0052	0.0814	0.061*	
H4B	0.2577	0.1524	0.0398	0.061*	
C5	0.3873 (2)	0.1383 (2)	0.14149 (15)	0.0381 (5)	
C6	0.3614 (2)	0.1462 (2)	0.26230 (14)	0.0337 (5)	
C7	0.4681 (2)	0.2063 (2)	0.29943 (14)	0.0354 (5)	
H7	0.5748	0.1561	0.2636	0.042*	
C8	0.4297 (2)	0.1905 (2)	0.42567 (15)	0.0367 (5)	
C9	0.5320 (3)	0.2232 (2)	0.47906 (17)	0.0471 (6)	
C10	0.4874 (3)	0.2148 (3)	0.60591 (17)	0.0525 (6)	
H10A	0.5232	0.2833	0.6315	0.063*	
H10B	0.5411	0.1279	0.6263	0.063*	
C11	0.3143 (3)	0.2328 (2)	0.66833 (16)	0.0451 (6)	
C12	0.2551 (3)	0.1381 (2)	0.61580 (15)	0.0427 (5)	
H12A	0.2915	0.0464	0.6383	0.051*	
H12B	0.1429	0.1598	0.6446	0.051*	
C13	0.3068 (2)	0.1463 (2)	0.49008 (14)	0.0349 (5)	
C14	0.0234 (3)	0.2601 (3)	0.2129 (2)	0.0638 (7)	
H14A	0.0834	0.3068	0.2375	0.096*	
H14B	−0.0758	0.2663	0.2698	0.096*	
H14C	0.0094	0.3002	0.1443	0.096*	
C15	0.0135 (4)	0.0391 (3)	0.1523 (2)	0.0787 (9)	
H15A	−0.0879	0.0488	0.2070	0.118*	
H15B	0.0644	−0.0547	0.1429	0.118*	
H15C	0.0047	0.0765	0.0817	0.118*	
C16	0.2298 (3)	0.3783 (3)	0.6599 (2)	0.0667 (7)	
H16A	0.2408	0.4010	0.5825	0.100*	
H16B	0.2731	0.4365	0.6893	0.100*	
H16C	0.1220	0.3895	0.7026	0.100*	
C17	0.2863 (3)	0.1950 (3)	0.79273 (17)	0.0620 (7)	
H17A	0.3218	0.2549	0.8271	0.093*	
H17B	0.3419	0.1041	0.7987	0.093*	
H17C	0.1776	0.2025	0.8303	0.093*	
C19	0.4554 (2)	0.3532 (2)	0.26216 (15)	0.0356 (5)	
C20	0.5677 (2)	0.3897 (2)	0.16956 (15)	0.0386 (5)	
H20	0.6574	0.3259	0.1343	0.046*	
C21	0.5475 (2)	0.5199 (2)	0.12944 (15)	0.0395 (5)	
C22	0.4165 (2)	0.6183 (2)	0.18321 (16)	0.0401 (5)	
C23	0.3081 (2)	0.5825 (2)	0.27940 (16)	0.0401 (5)	
C24	0.3262 (2)	0.4511 (2)	0.31724 (15)	0.0402 (5)	
H24	0.2510	0.4279	0.3804	0.048*	
C25	0.7846 (3)	0.4710 (3)	−0.02256 (19)	0.0637 (7)	
H25A	0.8480	0.4413	0.0246	0.096*	
H25B	0.8412	0.5123	−0.0894	0.096*	
H25C	0.7565	0.3952	−0.0428	0.096*	
C26	0.1124 (3)	0.6717 (3)	0.44614 (19)	0.0619 (7)	
H26A	0.0458	0.6106	0.4588	0.093*	
H26B	0.0524	0.7580	0.4775	0.093*	
H26C	0.1899	0.6371	0.4815	0.093*	
O1	0.21042 (15)	0.10108 (15)	0.44842 (10)	0.0387 (4)	
O2	0.50511 (17)	0.16061 (16)	0.07249 (11)	0.0510 (4)	
O3	0.6537 (2)	0.2498 (2)	0.42231 (14)	0.0770 (6)	
O4	0.18392 (19)	0.68547 (17)	0.33083 (12)	0.0575 (5)	
O5	0.39109 (19)	0.74774 (16)	0.14607 (12)	0.0555 (5)	
H5	0.4487	0.7521	0.0813	0.073 (9)*	
O6	0.64868 (18)	0.56614 (16)	0.03654 (12)	0.0530 (4)	

### Atomic displacement parameters (Å^2^)

**Table:**

	*U*^11^	*U*^22^	*U*^33^	*U*^12^	*U*^13^	*U*^23^
C1	0.0421 (11)	0.0337 (13)	0.0199 (9)	−0.0064 (9)	−0.0053 (8)	0.0003 (7)
C2	0.0538 (13)	0.0461 (15)	0.0271 (10)	−0.0215 (11)	−0.0090 (9)	0.0029 (9)
C3	0.0586 (14)	0.0583 (17)	0.0297 (11)	−0.0259 (13)	−0.0163 (10)	0.0083 (9)
C4	0.0692 (15)	0.0593 (17)	0.0239 (10)	−0.0193 (13)	−0.0115 (10)	−0.0005 (9)
C5	0.0487 (12)	0.0331 (13)	0.0237 (9)	−0.0060 (10)	−0.0028 (9)	−0.0005 (8)
C6	0.0393 (11)	0.0327 (12)	0.0232 (9)	−0.0061 (9)	−0.0047 (8)	0.0018 (7)
C7	0.0339 (10)	0.0413 (13)	0.0235 (9)	−0.0062 (9)	−0.0024 (8)	0.0024 (8)
C8	0.0402 (11)	0.0398 (14)	0.0252 (9)	−0.0066 (10)	−0.0068 (8)	0.0025 (8)
C9	0.0458 (13)	0.0583 (17)	0.0372 (11)	−0.0162 (12)	−0.0113 (10)	0.0029 (10)
C10	0.0585 (14)	0.0681 (18)	0.0382 (12)	−0.0219 (13)	−0.0209 (11)	0.0044 (10)
C11	0.0577 (14)	0.0515 (16)	0.0275 (10)	−0.0153 (12)	−0.0139 (9)	0.0006 (9)
C12	0.0507 (13)	0.0514 (15)	0.0238 (9)	−0.0152 (11)	−0.0083 (9)	0.0050 (9)
C13	0.0396 (11)	0.0395 (13)	0.0230 (9)	−0.0082 (9)	−0.0081 (8)	0.0028 (8)
C14	0.0564 (15)	0.067 (2)	0.0646 (16)	−0.0124 (14)	−0.0213 (13)	0.0180 (13)
C15	0.103 (2)	0.117 (3)	0.0458 (14)	−0.067 (2)	−0.0367 (15)	0.0168 (14)
C16	0.0893 (19)	0.054 (2)	0.0497 (14)	−0.0081 (15)	−0.0184 (13)	−0.0075 (12)
C17	0.0836 (18)	0.080 (2)	0.0302 (11)	−0.0318 (16)	−0.0193 (12)	0.0020 (11)
C19	0.0387 (11)	0.0428 (14)	0.0248 (9)	−0.0140 (10)	−0.0070 (8)	0.0025 (8)
C20	0.0378 (11)	0.0470 (15)	0.0287 (10)	−0.0140 (10)	−0.0055 (8)	0.0012 (9)
C21	0.0462 (12)	0.0481 (15)	0.0247 (10)	−0.0206 (11)	−0.0064 (9)	0.0047 (9)
C22	0.0519 (13)	0.0386 (14)	0.0315 (10)	−0.0147 (11)	−0.0140 (9)	0.0054 (9)
C23	0.0441 (12)	0.0416 (15)	0.0308 (10)	−0.0077 (10)	−0.0088 (9)	−0.0005 (9)
C24	0.0423 (12)	0.0449 (15)	0.0264 (10)	−0.0136 (10)	−0.0009 (8)	0.0045 (8)
C25	0.0559 (15)	0.077 (2)	0.0435 (13)	−0.0255 (14)	0.0073 (11)	0.0093 (12)
C26	0.0513 (14)	0.066 (2)	0.0489 (14)	0.0000 (13)	0.0002 (11)	−0.0063 (12)
O1	0.0433 (8)	0.0533 (10)	0.0188 (6)	−0.0185 (7)	−0.0048 (6)	0.0037 (6)
O2	0.0561 (9)	0.0598 (12)	0.0250 (7)	−0.0165 (8)	0.0044 (7)	0.0004 (6)
O3	0.0588 (11)	0.1335 (19)	0.0502 (10)	−0.0510 (12)	−0.0144 (9)	0.0094 (10)
O4	0.0634 (10)	0.0447 (11)	0.0443 (9)	0.0036 (8)	−0.0047 (8)	0.0056 (7)
O5	0.0733 (11)	0.0444 (11)	0.0366 (9)	−0.0133 (9)	−0.0053 (8)	0.0110 (7)
O6	0.0582 (10)	0.0533 (11)	0.0370 (8)	−0.0234 (8)	0.0034 (7)	0.0084 (7)

### Geometric parameters (Å, º)

**Table:**

C1—C6	1.330 (2)	C14—H14A	0.9600
C1—O1	1.377 (2)	C14—H14B	0.9600
C1—C2	1.486 (3)	C14—H14C	0.9600
C2—C3	1.534 (3)	C15—H15A	0.9600
C2—H2A	0.9700	C15—H15B	0.9600
C2—H2B	0.9700	C15—H15C	0.9600
C3—C14	1.518 (3)	C16—H16A	0.9600
C3—C15	1.524 (3)	C16—H16B	0.9600
C3—C4	1.525 (3)	C16—H16C	0.9600
C4—C5	1.498 (3)	C17—H17A	0.9600
C4—H4A	0.9700	C17—H17B	0.9600
C4—H4B	0.9700	C17—H17C	0.9600
C5—O2	1.218 (2)	C19—C20	1.383 (2)
C5—C6	1.466 (2)	C19—C24	1.386 (3)
C6—C7	1.511 (3)	C20—C21	1.376 (3)
C7—C8	1.513 (2)	C20—H20	0.9300
C7—C19	1.525 (3)	C21—O6	1.373 (2)
C7—H7	0.9800	C21—C22	1.394 (3)
C8—C13	1.331 (3)	C22—O5	1.358 (2)
C8—C9	1.460 (3)	C22—C23	1.388 (3)
C9—O3	1.213 (2)	C23—C24	1.378 (3)
C9—C10	1.512 (3)	C23—O4	1.379 (3)
C10—C11	1.527 (3)	C24—H24	0.9300
C10—H10A	0.9700	C25—O6	1.419 (3)
C10—H10B	0.9700	C25—H25A	0.9600
C11—C16	1.520 (3)	C25—H25B	0.9600
C11—C12	1.523 (3)	C25—H25C	0.9600
C11—C17	1.532 (3)	C26—O4	1.394 (3)
C12—C13	1.495 (2)	C26—H26A	0.9600
C12—H12A	0.9700	C26—H26B	0.9600
C12—H12B	0.9700	C26—H26C	0.9600
C13—O1	1.374 (2)	O5—H5	0.8200
			
C6—C1—O1	122.65 (17)	O1—C13—C12	110.98 (15)
C6—C1—C2	125.82 (16)	C3—C14—H14A	109.5
O1—C1—C2	111.53 (15)	C3—C14—H14B	109.5
C1—C2—C3	111.07 (16)	H14A—C14—H14B	109.5
C1—C2—H2A	109.4	C3—C14—H14C	109.5
C3—C2—H2A	109.4	H14A—C14—H14C	109.5
C1—C2—H2B	109.4	H14B—C14—H14C	109.5
C3—C2—H2B	109.4	C3—C15—H15A	109.5
H2A—C2—H2B	108.0	C3—C15—H15B	109.5
C14—C3—C15	109.6 (2)	H15A—C15—H15B	109.5
C14—C3—C4	111.30 (18)	C3—C15—H15C	109.5
C15—C3—C4	109.35 (19)	H15A—C15—H15C	109.5
C14—C3—C2	109.76 (18)	H15B—C15—H15C	109.5
C15—C3—C2	109.56 (18)	C11—C16—H16A	109.5
C4—C3—C2	107.25 (18)	C11—C16—H16B	109.5
C5—C4—C3	116.38 (16)	H16A—C16—H16B	109.5
C5—C4—H4A	108.2	C11—C16—H16C	109.5
C3—C4—H4A	108.2	H16A—C16—H16C	109.5
C5—C4—H4B	108.2	H16B—C16—H16C	109.5
C3—C4—H4B	108.2	C11—C17—H17A	109.5
H4A—C4—H4B	107.3	C11—C17—H17B	109.5
O2—C5—C6	120.52 (19)	H17A—C17—H17B	109.5
O2—C5—C4	120.89 (17)	C11—C17—H17C	109.5
C6—C5—C4	118.57 (17)	H17A—C17—H17C	109.5
C1—C6—C5	117.83 (18)	H17B—C17—H17C	109.5
C1—C6—C7	123.52 (16)	C20—C19—C24	119.02 (19)
C5—C6—C7	118.64 (16)	C20—C19—C7	120.75 (18)
C6—C7—C8	109.05 (15)	C24—C19—C7	120.14 (16)
C6—C7—C19	110.37 (15)	C21—C20—C19	120.34 (19)
C8—C7—C19	112.14 (16)	C21—C20—H20	119.8
C6—C7—H7	108.4	C19—C20—H20	119.8
C8—C7—H7	108.4	O6—C21—C20	125.29 (19)
C19—C7—H7	108.4	O6—C21—C22	113.83 (19)
C13—C8—C9	118.53 (17)	C20—C21—C22	120.88 (18)
C13—C8—C7	122.46 (18)	O5—C22—C23	118.68 (19)
C9—C8—C7	119.01 (17)	O5—C22—C21	122.90 (18)
O3—C9—C8	120.50 (19)	C23—C22—C21	118.42 (19)
O3—C9—C10	120.9 (2)	C24—C23—O4	123.88 (18)
C8—C9—C10	118.48 (18)	C24—C23—C22	120.51 (19)
C9—C10—C11	115.06 (18)	O4—C23—C22	115.60 (19)
C9—C10—H10A	108.5	C23—C24—C19	120.71 (18)
C11—C10—H10A	108.5	C23—C24—H24	119.6
C9—C10—H10B	108.5	C19—C24—H24	119.6
C11—C10—H10B	108.5	O6—C25—H25A	109.5
H10A—C10—H10B	107.5	O6—C25—H25B	109.5
C16—C11—C12	110.94 (19)	H25A—C25—H25B	109.5
C16—C11—C10	110.21 (19)	O6—C25—H25C	109.5
C12—C11—C10	107.87 (18)	H25A—C25—H25C	109.5
C16—C11—C17	108.95 (19)	H25B—C25—H25C	109.5
C12—C11—C17	108.65 (18)	O4—C26—H26A	109.5
C10—C11—C17	110.20 (19)	O4—C26—H26B	109.5
C13—C12—C11	112.93 (17)	H26A—C26—H26B	109.5
C13—C12—H12A	109.0	O4—C26—H26C	109.5
C11—C12—H12A	109.0	H26A—C26—H26C	109.5
C13—C12—H12B	109.0	H26B—C26—H26C	109.5
C11—C12—H12B	109.0	C13—O1—C1	118.10 (14)
H12A—C12—H12B	107.8	C23—O4—C26	116.45 (17)
C8—C13—O1	123.71 (16)	C22—O5—H5	109.5
C8—C13—C12	125.31 (18)	C21—O6—C25	116.86 (18)
			
C6—C1—C2—C3	−30.4 (3)	C10—C11—C12—C13	−48.2 (2)
O1—C1—C2—C3	150.19 (18)	C17—C11—C12—C13	−167.64 (19)
C1—C2—C3—C14	−69.1 (2)	C9—C8—C13—O1	−173.90 (19)
C1—C2—C3—C15	170.5 (2)	C7—C8—C13—O1	5.3 (3)
C1—C2—C3—C4	51.9 (2)	C9—C8—C13—C12	5.7 (3)
C14—C3—C4—C5	71.9 (2)	C7—C8—C13—C12	−175.1 (2)
C15—C3—C4—C5	−166.9 (2)	C11—C12—C13—C8	22.5 (3)
C2—C3—C4—C5	−48.2 (3)	C11—C12—C13—O1	−157.83 (18)
C3—C4—C5—O2	−162.6 (2)	C6—C7—C19—C20	−102.2 (2)
C3—C4—C5—C6	19.2 (3)	C8—C7—C19—C20	136.03 (18)
O1—C1—C6—C5	177.92 (18)	C6—C7—C19—C24	74.3 (2)
C2—C1—C6—C5	−1.4 (3)	C8—C7—C19—C24	−47.5 (2)
O1—C1—C6—C7	−3.6 (3)	C24—C19—C20—C21	−3.3 (3)
C2—C1—C6—C7	177.01 (19)	C7—C19—C20—C21	173.18 (18)
O2—C5—C6—C1	−170.53 (19)	C19—C20—C21—O6	−177.93 (18)
C4—C5—C6—C1	7.7 (3)	C19—C20—C21—C22	2.2 (3)
O2—C5—C6—C7	11.0 (3)	O6—C21—C22—O5	0.7 (3)
C4—C5—C6—C7	−170.83 (19)	C20—C21—C22—O5	−179.44 (18)
C1—C6—C7—C8	7.4 (3)	O6—C21—C22—C23	−178.83 (17)
C5—C6—C7—C8	−174.20 (17)	C20—C21—C22—C23	1.1 (3)
C1—C6—C7—C19	−116.2 (2)	O5—C22—C23—C24	177.31 (19)
C5—C6—C7—C19	62.2 (2)	C21—C22—C23—C24	−3.2 (3)
C6—C7—C8—C13	−8.1 (3)	O5—C22—C23—O4	−1.7 (3)
C19—C7—C8—C13	114.5 (2)	C21—C22—C23—O4	177.83 (18)
C6—C7—C8—C9	171.07 (18)	O4—C23—C24—C19	−179.03 (18)
C19—C7—C8—C9	−66.4 (2)	C22—C23—C24—C19	2.1 (3)
C13—C8—C9—O3	172.1 (2)	C20—C19—C24—C23	1.2 (3)
C7—C8—C9—O3	−7.1 (3)	C7—C19—C24—C23	−175.31 (18)
C13—C8—C9—C10	−4.4 (3)	C8—C13—O1—C1	−0.4 (3)
C7—C8—C9—C10	176.45 (19)	C12—C13—O1—C1	179.94 (16)
O3—C9—C10—C11	158.3 (2)	C6—C1—O1—C13	−0.5 (3)
C8—C9—C10—C11	−25.3 (3)	C2—C1—O1—C13	178.95 (17)
C9—C10—C11—C16	−70.9 (3)	C24—C23—O4—C26	27.8 (3)
C9—C10—C11—C12	50.4 (3)	C22—C23—O4—C26	−153.2 (2)
C9—C10—C11—C17	168.8 (2)	C20—C21—O6—C25	0.3 (3)
C16—C11—C12—C13	72.6 (2)	C22—C21—O6—C25	−179.86 (19)

### Hydrogen-bond geometry (Å, º)

**Table:**

*D*—H···*A*	*D*—H	H···*A*	*D*···*A*	*D*—H···*A*
O5—H5···O2^i^	0.82	2.02	2.762 (2)	151

Symmetry code: (i) −*x*+1, −*y*+1, −*z*.
